# Investigating the Cracking Processes and Bearing Performance of Fissured Concrete SCB Specimens via DEM-Based Mesoscopic Modeling Considering Fissure Angle, Aggregate Content and Porosity

**DOI:** 10.3390/ma18225140

**Published:** 2025-11-12

**Authors:** Qinrong Li, Suyi Liu, Yifei Li, Mingyue Qiu, Ruitong Zhang, Cheng Chen, Shuyang Yu

**Affiliations:** 1 Transport Planning and Research Institute, Ministry of Transport, Beijing 100028, China; liqinrong_beijing@163.com; 2 School of Architectural and Hydraulic Engineering, Jiujiang Polytechnic University of Science and Technology, Jiujiang 332020, China; liu06014@163.com; 3 Department of Mechanical Engineering, Huzhou University, Huzhou 313002, China; 4 School of Transportation and Civi Engineering, Nantong University, Nantong 226019, Chinazhangrt257@163.com (R.Z.); 5 College of Mechanics and Engineering Science, Hohai University, Nanjing 210098, China

**Keywords:** fissured concrete, discrete element method, semi-circular bending specimens, crack propagation, mesoscopic fracture mechanism

## Abstract

To reveal the mesoscopic fracture mechanism of fissured concrete, this study employed the discrete element method (DEM) and adopted the parallel bond model (PBM) within the two-dimensional particle flow code (PFC2D) to construct a mesoscopic model of concrete semi-circular bending (SCB) specimens with prefabricated fissures. Three sets of schemes were designed by varying prefabricated fissure angles (0–45°), aggregate contents (30–45%), and porosities (3–6%), and numerical simulations of three-point bending loads were conducted to explore the effects of each parameter on the crack propagation law and load-bearing performance of the specimens. Validation was performed by comparing the simulated load–displacement curves with the typical quasi-brittle mechanical characteristics of concrete (exhibiting “linear elastic rise–pre-peak stress fluctuation–nonlinear decline”) and verifying that the DEM could accurately capture the entire process from microcrack initiation at the aggregate–mortar interface, crack deflection/bifurcation induced by pores, to macroscopic fracture penetration—consistent with the known mesoscopic damage evolution law of concrete. The results indicate that the crack propagation mode evolves from straight extension to tortuous branching as parameters change. Moreover, the peak strength first increases and then decreases with the increase in each parameter: when the fissure angle is 15°, the aggregate content is 35%, and the porosity is 4%, the specimens achieve an optimal balance between crack propagation resistance and energy dissipation, resulting in the best load-bearing performance. Specifically, the prefabricated fissure angle dominates the stress type (tension–shear transition); aggregates regulate crack resistance through a “blocking–diverting” effect; and pores, acting as defects, influence stress concentration. This study verifies the reliability of DEM in simulating concrete fracture behavior, enriches the mesoscopic fracture theory of concrete, and provides reliable references for the optimization of concrete material proportioning (e.g., aggregate–porosity ratio adjustment) and anti-cracking design of infrastructure (e.g., pavement, tunnel linings) in engineering practices.

## 1. Introduction

Concrete, as one of the most widely used construction materials in the field of civil engineering, is essentially a quasi-brittle material with internal microcracks and pores [1,2,3]. During long-term service, under the combined effects of external loads (such as traffic loads and structural self-weight), environmental factors (such as temperature changes and freeze–thaw cycles), and material aging, these internal microcracks tend to initiate, propagate, and coalesce, eventually leading to macroscopic cracking in concrete structures. Once crack propagation occurs inside concrete, it not only directly damages the integrity of the material’s internal structure, resulting in a significant decline in key mechanical properties such as strength, stiffness, and durability [4,5], but also provides penetration channels for harmful substances like chloride ions and sulfate ions. This accelerates steel corrosion and the weathering of internal aggregates, further exacerbating structural damage. Such damage gradually spreads to critical infrastructure, including bridges, tunnels, dams, and highway pavements, seriously endangering their safety and stability during service. It may lead to issues such as insufficient structural load-bearing capacity, a substantial reduction in service life, and even catastrophic accidents like collapses [6,7]. Therefore, conducting research on the fracture mechanism of concrete is of great significance.

Research on the crack propagation mechanism of concrete is mainly divided into experimental research, theoretical research, and numerical simulation research. Experimental research is the fundamental means to explore the cracking characteristics of concrete. It can directly obtain the macroscopic manifestations of concrete cracks in real service scenarios (such as crack propagation paths, ultimate load, and fracture toughness). The measured data can intuitively reflect the actual mechanical behavior of the material, providing a reliable benchmark for the establishment and verification of numerical simulation models. For example, Yu et al. [8] explored the effects of grouted effects of cement on the fracture properties of grouted bodies; Cai et al. [9] focused on the insufficient toughness of manufactured sand concrete (MSC) and studied the effects of notch depth ratio and steel fiber volume fraction on its Mode II fracture performance through double-notched cube (DNC) tests combined with DIC technology. However, the observation range of concrete experimental research is limited. Traditional experiments mostly rely on surface observation methods, making it difficult to capture the initiation, propagation, and interaction processes of internal microcracks in concrete. This inability to fully reveal the microscopic mechanism of crack evolution may lead to deviations in the understanding of the essence of cracking.

Compared with traditional experimental testing, numerical simulation of concrete fracture shows significant advantages in exploring the concrete cracking mechanism and supporting engineering design: First, it has strong observability of microscopic mechanisms. It can break through the limitations of experiments in monitoring internal cracks, directly capture the entire process from microcrack initiation and propagation to macroscopic coalescence through modeling, and clearly present microscopic behaviors such as the bond failure at the aggregate–mortar interface and the evolution of microcracks inside the mortar. This provides an intuitive basis for revealing the intrinsic mechanism of concrete fracture. Second, it has high efficiency in parametric research. Without repeatedly preparing specimens and adjusting experimental equipment, it can quickly carry out comparative studies of multiple variables and working conditions by modifying model parameters (such as the geometric shape of prefabricated fissures, aggregate gradation, load conditions, and environmental parameters). This systematically analyzes the influence laws of various factors on concrete fracture behavior and greatly reduces experimental costs and time consumption. According to differences in modeling principles and applicable scenarios, numerical simulation methods for concrete fracture can be divided into two categories: (1) continuous medium-based methods; (2) discrete medium-based methods. Continuous medium methods are based on the assumption of material continuity and describe macroscopic mechanical behavior by solving governing equations, which are suitable for the initial stage of crack initiation and macroscopic fracture analysis. They mainly include the finite element method (FEM), finite difference method (FDM), and extended finite element method (XFEM). The finite element method can effectively simulate nonlinear deformation and energy dissipation during the fracture process by introducing special constitutive models (such as damage and plastic models) and separation/embedding techniques. In addition, this method can conveniently handle complex geometric shapes and boundary conditions and is efficiently integrated with mature commercial software platforms, providing a practical framework for fracture analysis of large-scale concrete structures. For example, Yao et al. [10] explored the rate-dependent fracture behavior of the concrete-rock interface under different roughness and strain rates through direct tensile and three-point bending tests combined with FEM. Teng et al. [11] proposed a random heterogeneous finite element modeling algorithm considering gradation and voids to study the fracture characteristics of asphalt concrete single-edge notched beams (SENB) at different temperatures; Zitouni et al. [12] developed a UMAT user subroutine in Fortran 90 language based on ABAQUS to solve the common mesh dependence problem in the finite element analysis of recycled aggregate concrete (RAC) structures and proposed an isotropic concrete damage model with tension–compression fracture energy regularization. Liu et al. [13] used FEM to construct a mesoscopic model of concrete containing aggregates with real morphologies and studied the influence of aggregate shape (characterized by fractal dimension) on the fracture behavior of concrete under triaxial compression. Rashidi et al. [14] explored the effect of steel fibers on the fracture energy of concrete through a combination of experiments and FEM. Yadav et al. [15] conducted three-point bending numerical simulations on M25 and M60 grade steel fiber-reinforced concrete (SFRC) beams based on FEM. However, the core limitation of the finite element method in dealing with concrete fracture lies in its dependence on continuum mechanics. It is difficult to naturally describe the discontinuous propagation process of cracks, and the method is sensitive to mesh division and requires large computational resources. Meanwhile, it faces challenges in accurately simulating the complex softening behavior and fracture energy consumption mechanism of concrete. The core advantages of the finite difference method lie in its computational efficiency and ease of implementation. Based on direct differential approximation, the program implementation is relatively simple, and it has a fast calculation speed and low memory consumption when dealing with regular meshes. For anisotropic damage-based or simplified fracture models, the finite difference method can provide an efficient solution. For example, Zhou et al. [16] realized the numerical modeling of the split Hopkinson pressure bar (SHPB) device through the coupling technology of DEM and FDM. Zhou et al. [17] simulated the dynamic fracture behavior of specimens with central interface cracks by taking rock–concrete bimaterial Brazilian disks as the research object and using the coupling technology of FDM and DEM. Wu et al. [18] modeled the FRP jacket (FDM) and concrete specimens, respectively, through the coupling technology of FDM and DEM to simulate the axial compression behavior of FRP-confined concrete. However, the finite difference method also has obvious shortcomings in dealing with concrete fracture problems. Its core limitation is poor adaptability to complex geometric boundaries and irregular crack paths, and it is difficult to accurately describe the tortuous crack front using regular meshes. In addition, this method has low accuracy in dealing with material nonlinearity and stress singularity at the crack tip, and it is usually difficult to naturally satisfy the conservation laws in dynamic fracture, which limits its application in accurately simulating the fracture process. The extended finite element method can introduce enrichment functions, allowing cracks to propagate independently inside elements, thereby getting rid of dependence on meshes and eliminating the need for complex mesh redivision when cracks propagate. This method can describe the singularity at the crack tip with high accuracy and naturally simulate the random initiation and tortuous propagation paths of cracks. For example, Hang et al. [19] carried out numerical simulations through four-point bending and four-point shear tests combined with XFEM and the cohesive zone model (CZM). Mukhtar et al. [20] addressed the problem of scarce and insufficiently verified 3D-printed concrete (3DPC) fracture models and proposed a 3D fracture simulation framework based on the generalized/extended finite element method (G/XFEM). Zeng et al. [21] constructed a numerical model for mesoscopic crack propagation in concrete through FEM and XFEM based on macroscopic fracture parameters. Xiao et al. [22] explored the low-temperature fracture behavior of railway asphalt concrete at −10 °C through the combination of SCB tests and XFEM. Golewski et al. [23] constructed a 3D numerical model of compact shear specimens (CSS) with double virtual cracks using XFEM, simulated the crack propagation of concrete containing fly ash (FA) under Mode II fracture based on the maximum principal stress criterion, and verified the model accuracy through experiments. However, the extended finite element method still has some shortcomings in dealing with concrete fracture. Its calculation accuracy and stability strongly depend on the selection of enrichment functions, and it is challenging to accurately describe the complex mixed-mode crack tip stress field in concrete. In addition, this method introduces additional degrees of freedom, which deteriorates the condition number of the system equations and may affect the solution efficiency and convergence. Moreover, the numerical implementation is relatively complex when dealing with a large number of intersecting or branched cracks. The core representative algorithm of discrete medium methods is the discrete element method (DEM), which models concrete as a combination of aggregates, mortar particles, and interface elements. By calculating the contact forces, relative displacements, and bond fractures between particles, it directly simulates the initiation, propagation, and coalescence of microcracks. It is a core method for studying the microscopic fracture mechanism of concrete, and is particularly suitable for analyzing the cracking process of fissured concrete (such as SCB specimens). For example, Zhu et al. [24] explored the effects of different rubber contents (0–30%) and specimen sizes on the fracture characteristics of self-compacting rubberized concrete (SCRC) through three-point bending tests and DEM. Zhao et al. [25] used DEM to construct a three-dimensional model and studied the influence of each component of recycled aggregates (RA) (old aggregates OA, old mortar OM, old interfacial transition zone ITZ1, new–old mortar interface ITZ2) on the mechanical properties of recycled aggregate concrete (RAC). Xue et al. [26] proposed a heterogeneous fracture simulation method for asphalt concrete combining algorithm generation technology and DEM, constructed a two-dimensional model containing random aggregate structures and asphalt mastic through a new algorithm, and used a bilinear cohesive fracture model to describe crack initiation and propagation. Nitka et al. [27] combined DEM with X-ray micro-CT technology to construct a two-dimensional mesoscopic model containing aggregates, mortar, interfacial transition zones (ITZ), and steel bars, and studied the fracture process of over-reinforced reinforced concrete short beams without vertical reinforcement under three-point bending. Nitka et al. [28] combined DEM with X-ray micro-CT technology to construct a three-dimensional mesoscopic model of concrete containing aggregates, cement matrix, macroscopic pores, and interfacial transition zones (ITZ), and studied the progressive fracture process of notched beams under bending. Skarżyński et al. [29] used FEM and DEM, combined with the real concrete microstructure obtained by X-ray micro-CT and SEM, to construct a four-phase model containing aggregates, cement matrix, interfacial transition zones (ITZ), and air voids, and studied the fracture behavior of notched concrete beams under three-point bending. In view of the superior characteristics of DEM in simulating concrete fracture, this study adopts the discrete element method to simulate the concrete fracture process.

Despite the extensive application of DEM in concrete fracture research, existing studies mostly focus on the single-factor influence of fissure characteristics, aggregate properties, or internal defects, lacking systematic exploration of the coupled effects of multiple key parameters on crack propagation and bearing performance [30,31]. To fill this research gap, this study makes original contributions in two aspects: first, it innovatively conducts a collective analysis of three core factors—prefabricated fissure angle, aggregate content, and porosity—systematically revealing their synergistic regulation mechanism on the mesoscopic fracture behavior of concrete SCB specimens, including the evolution of crack propagation mode from straight extension to tortuous branching and the variation law of peak strength with parameter changes. Second, it optimizes the DEM-based mesoscopic modeling method for fissured concrete, verifying that DEM can accurately capture the entire process from microcrack initiation to macroscopic penetration under multi-parameter coupling [32]. The research results provide a reference for a comprehensive understanding of the mesoscopic fracture mechanism of concrete.

## 2. PFC Principles and Numerical Model

### 2.1. Parallel Bond Model (PBM)

In this study, DEM was employed to perform numerical simulations of the three-point bending test. The most prominent advantage of DEM lies in its ability to accurately characterize the discontinuous failure characteristics of rock-like materials during loading, as well as the multi-scale damage evolution law from the interaction between mesoscopic particles to the macroscopic overall instability. The two-dimensional particle flow code (PFC2D) was selected as the specific simulation tool in this research, and its core simulation logic is based on the assumption of discrete particle aggregates.

**Figure 1:**
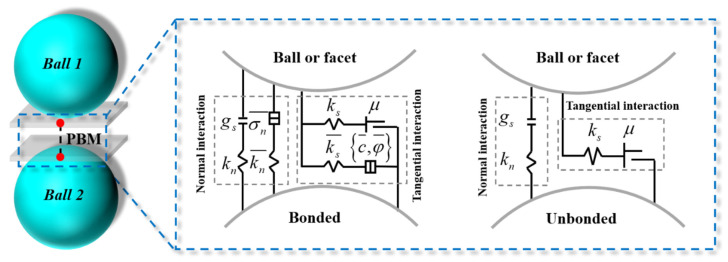
Schematic diagram of the parallel linear bond model.

The PBM was adopted for the simulation, and its principle is illustrated in Figure 1. This model incorporates an elastic cementation layer with a defined stiffness at the contact area between adjacent particles, which is used to equivalently represent the cementation effect between rock particles. After the model is initialized, the particles remain in a bonded state; relative movement between particles generates contact forces and moments. When the local stress exceeds the bond strength threshold, the bond breaks and induces cracks, and the particles transition to an unbonded state. At this stage, tension and friction are transmitted only through direct contact between particles, which is consistent with the mechanical response characteristics of rock materials after failure.

The PBM provides two contact interfaces: a frictional interface capable of transmitting damping forces and linear forces, and a bonded interface responsible for transmitting bonding forces and moments. The formulas for calculating the total contact force and moment are as follows:

(1) $$F_{c}=F^{l}+F^{d}+\bar{F}$$

(2) $$M_{c}=\bar{M}$$

where $F^{l}$ is the linear force, $F^{d}$ is the damping force, $\bar{F}$ is the bonding force, and $\bar{M}$ is the bonding contact moment. With the increase in external force, relative motion occurs between particles. When the tensile or shear stress at any point exceeds its ultimate strength, corresponding failure will occur.

### 2.2. Model Generation Method

As a typical multiphase composite material, the microstructure of concrete mainly consists of four components: mortar, aggregates, ITZ (interface transition zone), and pores. The composition and functions of each component collectively determine the macroscopic properties of concrete.

**Figure 2:**
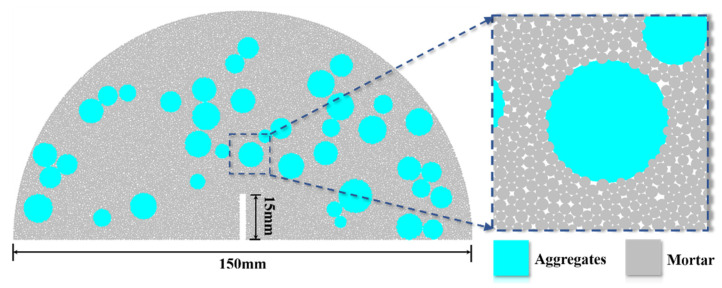
Schematic diagram of the numerical model.

The model generated by the PBM in this study is shown in Figure 2. The fracture part was achieved by the particle deletion method. Firstly, the outer frame of the fracture was imported externally to generate walls. After the particles were generated, the walls and the particles inside the frame were deleted. The fracture generated in this way has a regular and aesthetic shape, which is consistent with the structure of fractures in reality. In addition, the concrete aggregate particles in the model were realized by assigning different geometric and mechanical property parameters from the mortar particles.

Based on the above mesoscopic structure, a semi-circular model with a diameter of 150 mm was established. The prefabricated fracture had a length of 15 mm and a width of 2 mm. The loading method was three-point bending. Specifically, a loading plate was added above the model, and two loading bars were, respectively, added at two positions 50 mm away from the center of the circle at the bottom of the model. The loading bars were kept stationary, and a constant downward velocity of 0.05 mm/s was applied to the loading plate for loading. The specific mesoscopic parameters are listed in Table 1.

### 2.3. Calculation Schemes

To investigate the effects of different prefabricated fracture angles, aggregate contents, and porosities on the crack propagation law and strength of fractured concrete SCB specimens, three sets of schemes were designed: Scheme A, different prefabricated fracture angles *α* (where *α* is defined as the angle between the fracture and the vertical direction); Scheme B, different aggregate contents defined as *P*_agg_; and Scheme C, different porosities defined as *P*_por_. The specific schemes are shown in Table 2.

## 3. Analysis of Numerical Simulation Results

### 3.1. Crack Propagation Process

#### 3.1.1. Effect of Different Prefabricated Fracture Angles on Fractured SCB Concrete Models

**Figure 3:**
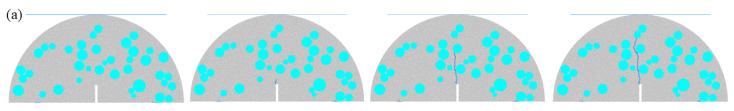

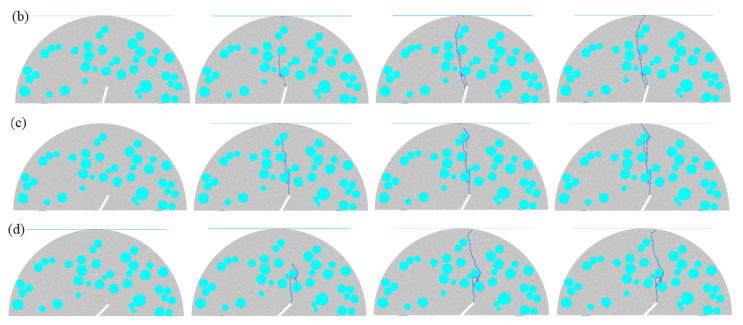
Crack propagation process of fractured SCB concrete models under different prefabricated fracture angles: (**a**) *α* = 0°; (**b**) *α* = 15°; (**c**) *α* = 30°; (**d**) *α* = 45°.

Figure 3 presents the crack propagation process of fractured SCB concrete models under different prefabricated fracture angles. By altering the stress field distribution inside the specimen, the prefabricated fracture angle significantly influences the crack initiation location, propagation path, and final fracture morphology. For Scheme A1, at the initial loading stage, the tip of the prefabricated fracture, which is in a state of pure tensile stress concentration (the fracture is completely aligned with the loading direction), first initiates a thin main crack. The initial direction of this crack coincides with the loading direction (vertically downward). As the load increases, the main crack continues to propagate toward the upper loading plate. When encountering aggregates along the way, the crack cannot directly penetrate the aggregates due to their higher elastic modulus and strength; instead, it detours along the aggregate–mortar interface transition zone. Specifically, the crack first deflects toward the edge of the aggregate, extends along the aggregate–mortar interface transition zone for a certain distance, and then returns to the vertical direction. During the detour process, only a small number of microcracks are generated inside the mortar, and the main crack path remains relatively straight overall. In the later loading stage, the main crack breaks through the obstruction of the last layer of aggregates and rapidly propagates through to the upper loading plate, forming a macroscopic main crack that penetrates the specimen vertically. Eventually, the specimen undergoes tension-dominated fracture, with a flat fracture surface perpendicular to the loading axis. For Scheme A2, due to the small-angle deviation between the fracture and the loading axis, the crack propagation mode is tensile–shear composite failure. At the initial loading stage, the first main crack initiates at the tip of the prefabricated fracture under the action of combined tensile–shear stress, with an initial propagation direction forming an angle of 5–8° with the loading axis. As the load continues to increase, a secondary crack initiates in the middle of the prefabricated fracture. After both the main crack and the secondary crack enter the stable propagation stage, they merge and continue to advance toward the upper loading plate. During this process, 2–3 aggregates are bypassed, and the deflection angle of the path gradually decreases (approaching the vertical direction). Finally, the crack rapidly propagates along the vertical direction and penetrates to the upper loading plate, resulting in a macroscopic crack morphology characterized by “bifurcation at the bottom and straightness in the upper-middle part”. For Scheme A3, as the fracture angle further increases, the proportion of the shear stress component rises significantly. At the initial loading stage, the main crack initiates at the tip of the prefabricated fracture under the action of high shear stress, with an initial propagation direction forming a larger angle with the loading axis. After the main crack bypasses two aggregates, bidirectional stress concentration occurs in the mortar area in front of the main crack due to the “stress dispersion effect” of the aggregates, leading to the initiation of a branched crack. As the load increases, the branched crack continues to propagate to the aggregate and stops. Subsequently, the main crack breaks through the obstruction of the last layer of aggregates at the top and penetrates to the loading plate, causing the model to fail. For Scheme A4, with the largest fracture angle, the interior of the specimen is dominated by shear stress. At the initial loading stage, a main crack initiates from the prefabricated fracture, with an initial propagation direction forming the largest angle with the loading axis. When the main crack propagates to the first aggregate and detours along the aggregate–mortar interface transition zone, a large number of microcracks are generated inside the mortar under high shear stress, directly resulting in the initiation of two branched cracks at the detour location. The cracks continue to propagate along the loading direction, bypassing 1 aggregate during the process. Each detour around an aggregate may be accompanied by small-sized bifurcations, and the two main branched cracks, along with multiple secondary branched cracks, form a dense crack network. When the load reaches the peak value, the main crack breaks through the obstruction of the last layer of aggregates at the top and penetrates to the loading plate, leading to the failure of the model.

#### 3.1.2. Effect of Different Aggregate Contents on Fractured SCB Concrete Models

**Figure 4:**
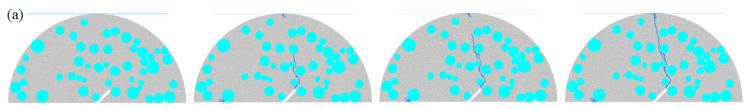

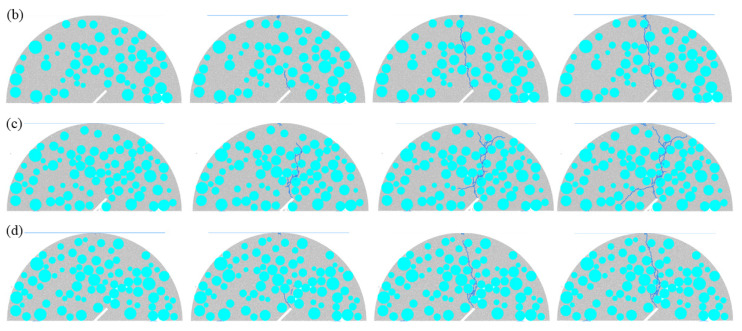
Crack propagation process of fractured SCB concrete models with different aggregate contents: (**a**) *P*_agg_ = 30%; (**b**) *P*_agg_ = 35%; (**c**) *P*_agg_ = 40%; (**d**) *P*_agg_ = 45%.

Figure 4 shows the crack propagation process of fractured SCB concrete models with different aggregate contents. By changing the heterogeneous distribution characteristics and mechanical property gradients inside concrete, the aggregate content (*P*_agg_) significantly affects the crack propagation resistance, path tortuosity, and fracture mode. For Scheme B1 (lowest aggregate content), the mortar matrix accounts for a large proportion. At the initial loading stage, due to the tensile stress state at the tip of the prefabricated fracture, a thin main crack first initiates, with an initial propagation direction consistent with the loading direction. After propagating for a certain distance, the main crack interacts with the aggregate closest to the prefabricated fracture, detours around the aggregate, and then extends to the top of the model, causing the model to fail. Notably, for Scheme B1, the crack propagation path is relatively straight due to the low aggregate content. For Scheme B2 (slightly increased aggregate content), the continuity of the mortar matrix decreases, and the crack propagation resistance enhances. The crack also initiates from the tip of the prefabricated fracture; after propagating for a certain distance, it extends along the edge of the nearest aggregate and then continues to propagate along the loading direction. During the propagation process, the crack interacts with aggregates, resulting in a relatively tortuous propagation path. For Scheme B3 (further increased aggregate content), the crack propagation resistance significantly improves. Due to the dense distribution of aggregates, after the crack initiates from the tip of the prefabricated fracture, it extends along the edges of the aggregates. Owing to the high aggregate content inside the specimen, the crack propagation path is highly tortuous and forms a large number of crack bifurcations. Finally, the crack extends to the top of the model, leading to the failure of the model. For Scheme B4 (highest aggregate content), the aggregates form a “dense distribution”, resulting in the maximum crack propagation resistance. After initiating from the prefabricated fracture, the crack immediately propagates along the aggregates. Eventually, it propagates along the pores between the aggregates and extends to the top of the model, causing the model to fail.

#### 3.1.3. Effect of Different Porosities on Fractured SCB Concrete Models

**Figure 5:**
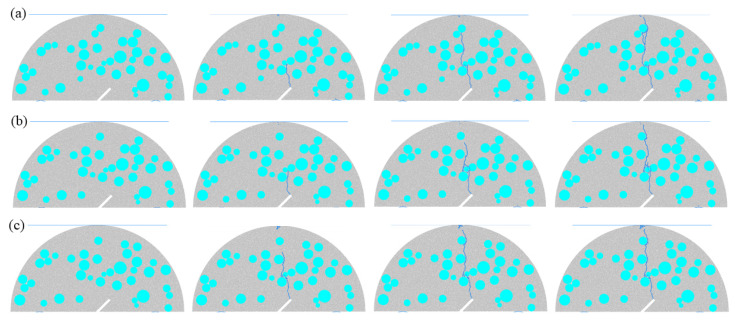

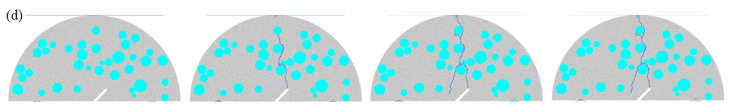
Crack propagation process of fractured SCB concrete models with different porosities: (**a**) *P*_por_ = 3%; (**b**) *P*_por_ = 4%; (**c**) *P*_por_ = 5%; (**d**) *P*_por_ = 6%.

Figure 5 illustrates the crack propagation process of fractured SCB concrete models with different porosities. As shown in the figure, porosity (Ppor), as a key internal defect parameter of concrete, significantly affects the crack initiation sensitivity, propagation path complexity, and fracture mode by altering the stress concentration distribution and material continuity. For Scheme C1 (lowest porosity), there are few internal defects and high material continuity. At the initial loading stage, due to stress concentration at the tip of the prefabricated fracture, a thin main crack first initiates, with an initial propagation direction basically coinciding with the loading direction. As the load increases, the crack propagates along the aggregates and finally extends to the top of the model, causing the model to fail. For Scheme C2 (slightly increased porosity), the number of pores increases, and the crack propagation path is similar to that of Scheme C1. However, more crack bifurcations appear during the propagation process. For Scheme C3 (significantly increased porosity), the pore distribution density is notably enhanced, resulting in a more tortuous crack propagation path. For Scheme C4 (highest porosity), the material continuity is extremely poor, and the crack path is not only tortuous but also accompanied by numerous bifurcations.

### 3.2. Load–Displacement Curves

**Figure 6:**
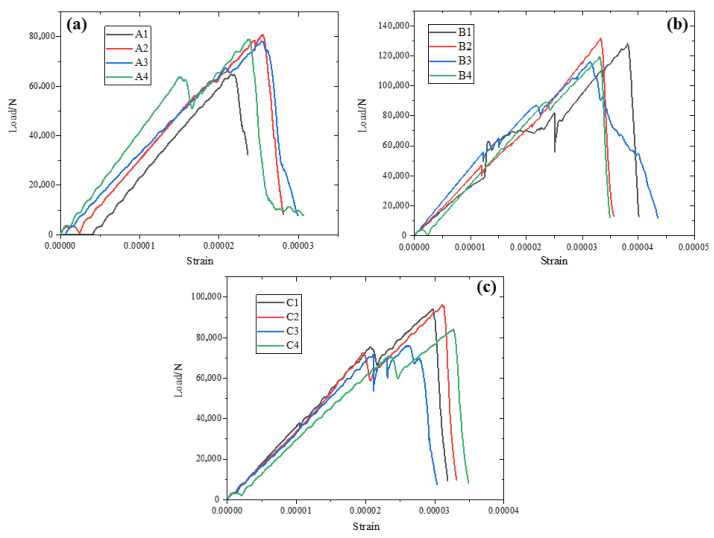
Load-strain curves: (**a**) Scheme A; (**b**) Scheme B; (**c**) Scheme C.

Figure 6 presents the law of load–displacement curves under different calculation schemes. Meanwhile, Table 3 presents the laws of peak load of different schemes. The load–displacement curves of all schemes exhibit the typical characteristics of quasi-brittle material fracture, namely “linear elastic rise—stress fluctuation before peak—nonlinear decline”. Moreover, the peak strength first increases and then decreases with the increase in variables (fracture angle, aggregate content, porosity). The curve shape is highly correlated with the crack propagation mode: the length of the linear segment increases with the increase in crack initiation resistance; the degree of fluctuation before the peak becomes more obvious with the increase in the number of crack bifurcations or secondary cracks; and the slope of the decline segment becomes steeper as the macroscopic fracture mode transitions from tension to shear or as the material continuity decreases. Essentially, different parameters affect the bearing capacity and energy dissipation characteristics of concrete by adjusting the stress concentration distribution and crack evolution path.

The law of load–displacement curves for Scheme A (different prefabricated fracture angles) fully corresponds to the evolution process of cracks from single straight extension to double-crack merging, then to branched extension, and finally to dense network formation. The peak strength first increases and then decreases with the increase in fracture angle, reaching the maximum when the fracture angle is 15°. Specifically, when the fracture angle is 0°, the linear elastic segment of the curve is the longest, and there is no obvious fluctuation before the peak. This is because after crack initiation, the crack extends straight along the loading axis without obvious stress release. After reaching the peak, the curve drops steeply without a plateau segment, corresponding to the pure tensile fracture caused by the rapid penetration of the main crack. When the fracture angle is 15°, the linear segment is slightly shorter than that in the 0° case, but several small stress fluctuations occur before the peak. These fluctuations are the result of local stress adjustment during the initiation and merging of double cracks. The peak strength is slightly improved because the double cracks jointly bear the load after merging, and the number of bypassed aggregates is moderate, achieving a balance between energy dissipation and bearing capacity. The slope of the decline segment after the peak becomes gentler, indicating that the resistance increases during the propagation of merged cracks, and the ductility is better than that in the 0° case. When the fracture angle is 30°, the linear segment is further shortened, and the frequency of stress fluctuations before the peak increases, corresponding to multiple stress releases during the branching of the main crack and the bypassing of aggregates. The peak strength decreases compared with that in the 15° case, because the increase in the proportion of shear stress leads to crack branching, resulting in scattered bearing paths. The decline segment after the peak exhibits multiple small stress drops, reflecting the stepwise instability caused by the gradual penetration of branched cracks. When the fracture angle is 45°, the linear segment is the shortest, and the load enters the nonlinear stage as soon as it reaches a part of the peak. The stress drops frequently and significantly before the peak, corresponding to the rapid evolution of the dense crack network. The peak strength decreases significantly because, under the dominance of shear stress, the number of cracks is large and the distribution is disordered, making it impossible to form an effective bearing path. The slope of the decline segment after the peak is the steepest, and the load drops sharply in a short time, corresponding to the shear instability caused by the instantaneous penetration of the crack network, which is consistent with the extension mode of a large number of branches formed after the crack bypasses aggregates.

The law of load–displacement curves for Scheme B (different aggregate contents) is highly correlated with the evolution process of cracks from straight extension, to slight tortuosity, then to multiple bifurcations, and finally to extension along pores. The peak strength first increases and then decreases with the increase in aggregate content, reaching the maximum when the aggregate content is moderately high. When the aggregate content is the lowest, the linear elastic segment of the curve is the longest, and there is no fluctuation before the peak. This is because the aggregates are sparse, and the crack extends straight along the mortar, resulting in low initiation resistance but a single bearing path. The peak strength is the lowest, and the curve drops steeply after the peak without a plateau segment, corresponding to the pure tensile fracture caused by the rapid penetration of the main crack and less energy dissipation. When the aggregate content is slightly higher, the linear segment is slightly shorter than that in the case of the lowest aggregate content, and several weak stress fluctuations occur before the peak, corresponding to local stress release when the crack bypasses a small number of aggregates. The peak strength is slightly improved because the increase in the number of aggregates enhances the crack bypass resistance, and a small number of secondary cracks do not disperse the main bearing path. The slope of the decline segment after the peak becomes gentler, and a short plateau appears, reflecting the increased energy consumption when the crack bypasses aggregates and the improved ductility. When the aggregate content is moderately high, the linear segment is the shortest, and the frequency and amplitude of stress fluctuations before the peak increase significantly. This corresponds to the process in which the crack bypasses in multiple directions along the edges of dense aggregates and initiates a large number of branched cracks; each fluctuation corresponds to the stress adjustment during the initiation of branched cracks. The peak strength is the highest because the aggregates form a semi-continuous distribution, which not only enhances the crack propagation resistance through the “blocking-diversion” effect but also avoids the concentration of interface transition zone defects due to excessive density. The decline segment after the peak presents a gentle slope and is accompanied by multiple small stress drops, corresponding to the stepwise instability caused by the gradual penetration of branched cracks, with the maximum energy dissipation and optimal ductility. When the aggregate content is the highest, the length of the linear segment is slightly longer than that in the case of moderately high aggregate content, but the amplitude of stress fluctuations before the peak decreases, corresponding to the crack extending along the gaps and pores between aggregates; although the path is tortuous, a large number of bifurcations are not observed. The peak strength decreases compared with that in the case of moderately high aggregate content, because the excessive density of aggregates leads to an increase in the total area of the interface transition zone, which becomes a stress-concentration weak area. Cracks preferentially extend along these areas, reducing the overall bearing capacity. The slope of the decline segment after the peak is slightly steeper than that in the case of moderately high aggregate content, reflecting the decreased crack propagation resistance caused by the damage of the interface transition zone, which is consistent with the mode in which cracks rapidly penetrate along the pores between aggregates.

The law of load–displacement curves for Scheme C (different porosities) is closely coupled with the evolution process of cracks from single penetration through pores, to slight bypassing of pores, then to multi-directional deflection, and finally to network penetration. The peak strength first increases and then decreases with the increase in porosity, reaching the maximum when the porosity is moderate. When the porosity is the lowest, the linear elastic segment of the curve is the longest, and there is no fluctuation before the peak. This is because the pores are sparse and independent, and after crack initiation, the crack extends along the loading axis and only needs to penetrate a small number of small pores without obvious stress release. The peak strength is relatively low, and the curve drops steeply after the peak without a plateau segment, corresponding to the pure tensile fracture caused by the rapid penetration of the main crack, with good material continuity but low crack propagation resistance. When the porosity is slightly higher, the linear segment is slightly shorter than that in the case of the lowest porosity, and a slight stress fluctuation occurs before the peak, corresponding to local stress adjustment when the crack penetrates medium-sized pores. The peak strength is slightly improved because the moderate number of pores promotes the formation of a stable bearing path for cracks through slight stress concentration, without destroying the material continuity, and a small number of secondary cracks do not disperse the main bearing capacity. The slope of the decline segment after the peak becomes gentler, and a short plateau appears, reflecting the increased energy dissipation when the crack penetrates pores and the slightly improved fracture ductility. When the porosity is moderate, the linear segment is further shortened, and the frequency of stress fluctuations before the peak increases, corresponding to the process in which the crack deflects in multiple directions along the “pore clusters” and extends with secondary cracks; each fluctuation corresponds to the stress release when the crack extends from one pore to another. The peak strength decreases compared with that in the case of slightly higher porosity, because the “pore clusters” lead to a decrease in material continuity, and cracks are prone to branching, resulting in scattered bearing paths. The decline segment after the peak exhibits multiple stress drops, corresponding to the stepwise instability caused by the gradual penetration of branched cracks along different pore paths. When the porosity is the highest, the linear segment is the shortest, and the load enters the nonlinear stage as soon as it reaches a part of the peak. The stress drops frequently and significantly before the peak, corresponding to the rapid evolution of the crack network formed by the connection of pores. The peak strength is the lowest because the large number of connected pores causes severe damage to material integrity, and cracks can rapidly penetrate without overcoming the resistance of the mortar. The slope of the decline segment after the peak is the steepest, and the load drops sharply in a short time, corresponding to the brittle fracture caused by the instantaneous penetration of the crack network, which is completely consistent with the mode in which cracks rapidly extend under the guidance of pores.

## 4. Discussion

### 4.1. Influence Mechanism of Different Fracture Angles, Aggregate Contents, and Porosities on the Initiation Mechanism of SCB Specimens

The influences of different fracture angles, aggregate contents, and porosities on the initiation mechanism of SCB specimens are essentially realized through the coupling effect of regulating the distribution characteristics (stress type, concentration degree) of the internal stress field of the specimens and the heterogeneous weak zones (mortar, interface transition zone (ITZ), pores) of the material. From the perspective of fracture angle, its core role is to change the type of dominant stress for crack initiation. When the angle is small (e.g., 0°), the prefabricated fracture is aligned with the loading direction, and the tip is dominated by pure tensile stress concentration. Crack initiation is completely controlled by the tensile strength of the mortar matrix, and only a single main crack initiates at the tip of the fracture. As the angle increases (e.g., 15–30°), the shear stress component is gradually superimposed to form a tensile–shear composite stress field, and the stress concentration area at the tip expands. In addition to the tip, the ITZ in the middle of the fracture (where stress superposition occurs due to aggregate obstruction) also becomes a potential initiation point, resulting in the initiation of double cracks. When the angle is the largest (e.g., 45°), shear stress dominates, and the stress field at the tip presents a multi-directional distribution. A large number of microdefects are generated inside the mortar under the action of shear stress, and crack initiation spreads from a single tip to multiple regions, forming the initial morphology of a dense crack network. From the perspective of aggregate content, it affects crack initiation by changing the stress dispersion effect and the distribution of weak zones. When the aggregate content is low, the mortar matrix is continuous and has low strength, so crack initiation is only dominated by the tensile stress concentration at the fracture tip and occurs inside the mortar. As the content increases to a moderate level, aggregates disperse the stress at the tip through the “blocking-diversion” effect, enhancing the resistance to crack initiation. Moreover, due to the uniform distribution of aggregates, the ITZ does not form concentrated defects, and crack initiation still mainly occurs at the tip but needs to overcome the bonding force of the ITZ. When the content is too high, the excessive concentration of aggregates leads to an increase in the total area of the ITZ, which becomes a concentrated weak zone. The stress at the tip is preferentially released at the ITZ, so the initiation location shifts from the mortar to the ITZ, and multi-crack initiation is likely to occur. From the perspective of porosity, as a built-in defect, it directly regulates the stress concentration points. At low porosity, pores are sparse and independent, causing little interference to the stress field at the tip, and crack initiation is still controlled by the tensile concentration at the tip. At moderate porosity, a small number of pores form local stress superposition near the tip, slightly increasing the sensitivity of crack initiation, but without damaging the stability of the stress field, and crack initiation is still a single main crack. When the porosity is too high, pores are connected to form “stress shortcuts”, and the stress at the tip is quickly transferred to the pore clusters. Stress concentration zones are formed simultaneously at the edges of multiple pores, so crack initiation changes from a single tip to collaborative initiation at multiple pores, and the initiation time is significantly advanced. Under the combined action of the three factors, the initiation mechanism of SCB specimens always follows the law of “matching between the dominant direction of the stress field and the location of the material weak zone”, that is, crack initiation preferentially occurs at the overlapping area of the “peak zone of dominant stress (tension/shear)” and the “zone with the lowest material strength (mortar/ITZ/pore)”.

### 4.2. Application Prospect of the Discrete Element Method in Simulating the Deterioration Process of Fractured Concrete

The discrete element method (DEM) shows significant application potential and broad prospects in simulating the deterioration process of fractured concrete. Its core advantage lies in breaking through the limitations of traditional experiments and continuous medium simulation, as it can capture the entire deterioration process with particle-level precision—from the initiation of microcracks at the aggregate–mortar interface (ITZ), the deflection and bifurcation of cracks induced by pores, to the penetration of macroscopic fractures. It clearly reveals the mesoscopic damage evolution mechanism of heterogeneous materials, and is particularly suitable for describing the progressive deterioration behavior of prefabricated fractured concrete under load, making up for the shortage that internal damage is difficult to observe in experiments. At the same time, DEM enables efficient parametric research. It can flexibly adjust key parameters such as fracture angle, aggregate gradation, and porosity to quickly carry out comparative analysis under multiple working conditions, providing an efficient tool for revealing the coupling effect of different factors on deterioration and optimizing the mix ratio of concrete materials. In the future, if DEM is further combined with experimental technologies such as X-CT and DIC for model verification, or integrated with the multi-field coupling effect of environmental factors (e.g., temperature, humidity), it will be able to more accurately simulate the deterioration process under actual service conditions. This will provide more reliable theoretical support for the safety assessment, service life prediction, and damage repair scheme formulation of infrastructure structures.

In constructing the discrete element method (DEM) model of this study, a simplified mesoscopic characterization scheme was adopted. The aggregate particle size was set to 5–15 mm (referring to the common aggregate gradation range of ordinary concrete in engineering), the pore size was controlled at 0.5–2 mm (matching the distribution characteristics of micro-pores in conventional concrete), and the interfacial transition zone (ITZ) was indirectly represented by assigning lower bond strength parameters to the aggregate–mortar interface (with the interface bond strength being approximately 70–80% of that of the mortar matrix), without independent subdivision of geometric and mechanical parameters for these three components. From the perspective of action mechanisms, aggregates alter the crack path through a “blocking-diversion” effect—larger-sized aggregates can force cracks to deflect more significantly, enhancing energy dissipation, while excessively small-sized aggregates exert weak resistance to cracks, easily leading to direct crack penetration through the mortar matrix. As inherent defects, pores’ size and distribution directly affect the degree of stress concentration: small-sized isolated pores have limited interference with crack propagation, whereas large-sized or interconnected pores tend to become crack initiation points, accelerating crack penetration. The ITZ, as a weak region inside concrete, exhibits degraded mechanical properties that reduce crack propagation resistance, causing cracks to preferentially extend along the aggregate–mortar interface. However, this study did not further quantify the influence of ITZ parameters (e.g., thickness, elastic modulus) on crack evolution. Restricted by current research conditions, this study employed a 2D DEM model, which cannot fully reflect the 3D spatial distribution characteristics of the concrete mesostructure. Future research will focus on three aspects: first, establishing a 3D mesoscopic model incorporating aggregates, ITZ, and pores to accurately characterize their geometric morphologies and spatial distributions; second, verifying the model using concrete’s real mesostructural data obtained via X-CT scanning to ensure microstructural accuracy; and third, systematically analyzing the coupled effects of parameters such as ITZ thickness, aggregate gradation, and pore connectivity on crack propagation, thereby further improving the mesoscopic characterization system of DEM in concrete fracture research.

From an engineering perspective, the research findings can be directly translated into practical applications: for pavement engineering, by optimizing the aggregate content (around 35%) and controlling porosity at 4% in concrete mixtures, combined with adjusting the prefabricated fissure angle (avoiding extreme angles like 45°) in pavement design, the crack propagation resistance of pavement concrete is significantly enhanced, reducing cracking caused by traffic loads and temperature cycles to achieve long-lasting pavement. For tunnel linings, leveraging the insight that a 15° fissure angle balances load-bearing capacity and energy dissipation, engineers can optimize the lining structure’s stress distribution, and by adopting the “blocking-diversion” effect of aggregates, design linings with reasonable aggregate gradation to inhibit the initiation and expansion of microcracks, improving the lining’s durability under long-term rock pressure; while achieving “never-fracturing” concrete is unrealistic due to material inherent characteristics, the study’s revelation of the coupled influence of key parameters on fracture behavior provides a scientific basis for anti-cracking design—for example, in bridge deck concrete, by precisely controlling porosity (4%) and aggregate content (35%), the material’s quasi-brittle performance is adjusted to delay macroscopic fracture, extending the structure’s service life and reducing maintenance costs.

## 5. Conclusions

(1) The prefabricated fracture angle exerts a significant regulatory effect on the crack propagation mode and bearing performance of fractured concrete SCB specimens, and the peak strength first increases and then decreases with the increase in the angle, reaching the optimal state when the angle is 15°. When the angle is 0°, the interior of the specimen is dominated by pure tensile stress, and the crack extends straight along the loading axis, eventually resulting in pure tensile fracture. At an angle of 15°, a tensile–shear composite stress field is formed, and the double cracks initiate and then merge for propagation. The number of bypassed aggregates is moderate, and the energy dissipation and bearing capacity reach a balance. When the angle ranges from 30° to 45°, the proportion of shear stress continues to increase, leading to more crack bifurcations and disordered paths, which fail to form an effective bearing path and thus result in the degradation of bearing performance.
(2) The aggregate content has a significant impact on the mechanical properties of SCB specimens by changing the internal heterogeneous characteristics of concrete and the crack propagation resistance. The peak strength first increases and then decreases with the increase in aggregate content, achieving the best performance when the content is 40%. When the aggregate content is 30%, the mortar matrix is continuous, and the crack propagation resistance is low, mainly resulting in pure tensile fracture. At a content of 35%, the increase in the number of aggregates enhances the crack bypass resistance, thereby improving the bearing performance. When the content reaches 40%, the aggregates form a semi-continuous distribution, which significantly enhances the crack propagation resistance through the “blocking-diversion” effect without the problem of concentrated defects in the interface transition zone (ITZ). At a content of 45%, the excessive aggregation of aggregates leads to an increase in the total area of the ITZ, and cracks preferentially propagate along the ITZ, resulting in a decrease in bearing performance.
(3) As a key internal defect parameter of concrete, porosity significantly affects the crack evolution and bearing capacity of SCB specimens. The peak strength first increases and then decreases with the increase in porosity, attaining the optimal value when the porosity is 4%. When the porosity is 3%, the pores are sparse and have little interference with the stress field at the crack tip, and the crack extends stably but with insufficient propagation resistance. At a porosity of 4%, a small number of pores promote the formation of a stable bearing path for cracks through slight stress concentration without damaging the material continuity. When the porosity ranges from 5% to 6%, the pores are connected to form “crack shortcuts”, and the multi-pore collaborative cracking leads to the rapid evolution of the crack network, which severely damages the material integrity and causes a significant decline in bearing capacity. This indicates that an appropriate level of porosity can optimize the bearing performance through stress adjustment, while an excessively high porosity will exacerbate deterioration.
(4) The discrete element method (DEM) demonstrates significant advantages and reliability in simulating the cracking process of fractured concrete SCB specimens, providing an efficient tool for the study of the mesoscopic fracture mechanism of concrete. Compared with traditional experiments, DEM can accurately capture the entire process from microcrack initiation and propagation to macroscopic penetration, clearly presenting mesoscopic behaviors such as the damage of the aggregate–mortar interface and pore-induced crack bifurcation. In contrast to the continuous medium method, it does not rely on the continuity assumption and can more truly reflect the influence of the heterogeneous characteristics of concrete on fracture. Meanwhile, DEM enables efficient parametric research, facilitating the rapid analysis of the coupling effect of multiple variables on fracture. The research results not only enrich the mesoscopic fracture theory of concrete but also provide theoretical support for the mix ratio optimization of concrete materials in infrastructure and safety assessment.
(5) This study has certain limitations that need to be addressed in subsequent work. Firstly, the current two-dimensional (2D) DEM model is used to construct the fissured concrete SCB specimens, which cannot fully reflect the three-dimensional (3D) spatial distribution characteristics of the concrete mesostructure. This may lead to deviations in the description of the 3D crack propagation path and the interaction of heterogeneous interfaces. Secondly, the interfacial transition zone (ITZ) in the model is characterized by an indirect assignment method (setting the interface bond strength to 70–80% of that of the mortar matrix), without independently subdividing the geometric parameters (e.g., thickness) and mechanical parameters (e.g., elastic modulus) of the ITZ. This makes it difficult to accurately quantify the regulatory mechanism of the ITZ on crack initiation and propagation. Finally, the numerical simulation does not introduce the coupling effect of environmental factors (such as temperature changes and freeze–thaw cycles) and loads, which cannot fully match the service scenarios of concrete structures in actual engineering. In subsequent studies, it is necessary to construct a 3D DEM model using real mesostructural data of concrete obtained by X-ray computed tomography (X-CT) scanning, and integrate multi-field coupling effects to further improve the engineering applicability of the simulation results.

## Figures and Tables

**Table 1:** Mesoscopic parameters [33].

Parameters of Aggregates		Parameters of Mortar	
Emod (Pa)	5.55 × 10^10^	Emod (Pa)	1.3 × 10^10^
Pb_emod (Pa)	5.55 × 10^10^	Pb_emod (Pa)	1.3 × 10^10^
Pb_ten (Pa)	2 × 10^7^	Pb_ten (Pa)	5 × 10^6^
Pb_coh (Pa)	2.5 × 10^7^	Pb_coh (Pa)	6 × 10^6^
Pb_fa (°)	40	Pb_fa (°)	45
Kratio	1.5	Kratio	2

**Table 2:** Calculation schemes.

Scheme	Details	Scheme	Details
A1	*α* = 0°	B1	*P*_agg_ = 30%
A2	*α* = 15°	B2	*P*_agg_ = 35%
A3	*α* = 30°	B3	*P*_agg_ = 40%
A4	*α* = 45°	B4	*P*_agg_ = 45%
C1	*P*_por_ = 3%	C2	*P*_por_ = 4%
C3	*P*_por_ = 5%	C4	*P*_por_ = 6%

**Table 3:** Peak loads in different schemes.

Scheme	Peak Load (N)	Scheme	Peak Load (N)
A1	6311	B1	128,311
A2	7912	B2	138,514
A3	7739	B3	117,662
A4	7814	B4	120,067
C1	9112	C2	9543
C3	7388	C4	8092

## Data Availability

The original contributions presented in this study are included in the article. Further inquiries can be directed to the corresponding authors.
